# Estradiol mitigates stress-induced cardiac injury and inflammation by downregulating ADAM17 via the GPER-1/PI3K signaling pathway

**DOI:** 10.1007/s00018-023-04886-6

**Published:** 2023-08-12

**Authors:** Joseph Adu-Amankwaah, Aisha Bushi, Rubin Tan, Adebayo Oluwafemi Adekunle, Gabriel Komla Adzika, Marie Louise Ndzie Noah, Iqra Nadeem, Seyram Yao Adzraku, Stephane Koda, Richard Mprah, Jie Cui, Kexue Li, Prosperl Ivette Wowui, Hong Sun

**Affiliations:** 1 Department of Physiology, Xuzhou Medical University, Xuzhou, 221004 Jiangsu China; 2 School of International Education, Xuzhou Medical University, Xuzhou, 221004 Jiangsu China; 3 Department of Neurobiology and Anatomy, Xuzhou Medical University, Xuzhou, 221004 Jiangsu China; 4 Department of Hematology, Key Laboratory of Bone Marrow Stem Cell, The Affiliated Hospital of Xuzhou Medical University, Xuzhou, 221002 China; 5 Department of Pathogenic Biology and Immunology, Xuzhou Medical University, Xuzhou, 221004 Jiangsu China

**Keywords:** Stress, Myocardial inflammation, Cardiac injury, ADAM17, Estradiol, GPER-1

## Abstract

Stress-induced cardiovascular diseases characterized by inflammation are among the leading causes of morbidity and mortality in postmenopausal women worldwide. Estradiol (E2) is known to be cardioprotective via the modulation of inflammatory mediators during stress. But the mechanism is unclear. TNFα, a key player in inflammation, is primarily converted to its active form by 'A Disintegrin and Metalloprotease 17' (ADAM17). We investigated if E2 can regulate ADAM17 during stress. Experiments were performed using female FVB wild-type (WT), C57BL/6 WT, and G protein-coupled estrogen receptor 1 knockout (GPER-1 KO) mice and H9c2 cells. The study revealed a significant increase in cardiac injury and inflammation during isoproterenol (ISO)-induced stress in ovariectomized (OVX) mice. Additionally, ADAM17’s membrane content (mADAM17) was remarkably increased in OVX and GPER-1 KO mice during stress. However, in vivo supplementation of E2 significantly reduced cardiac injury, mADAM17, and inflammation. Also, administering G1 (GPER-1 agonist) in mice under stress reduced mADAM17. Further experiments demonstrated that E2, via GPER-1/PI3K pathway, localized ADAM17 at the perinuclear region by normalizing β1AR-Gαs, mediating the switch from β2AR-Gαi to Gαs, and reducing phosphorylated kinases, including p38 MAPKs and ERKs. Thus, using G15 and LY294002 to inhibit GPER-1 and its down signaling molecule, PI3K, respectively, in the presence of E2 during stress resulted in the disappearance of E2’s modulatory effect on mADAM17. In vitro knockdown of ADAM17 during stress significantly reduced cardiac injury and inflammation, confirming its significant inflammatory role. These interesting findings provide novel evidence that E2 and G1 are potential therapeutic agents for ADAM17-induced inflammatory diseases associated with postmenopausal females.

## Introduction

Stress-induced cardiovascular diseases are among the leading causes of morbidity and mortality in postmenopausal women worldwide. Following menopause, the risk of cardiovascular diseases (CVDs) skyrockets [1], accounting for approximately one-third of all deaths in aged women [2]. Therefore, elucidating the mechanisms involved in preventing the development of CVDs in postmenopausal women under stressful conditions remains a major research goal. According to epidemiological studies, changes in female reproductive hormones, predominantly estradiol (E2) declination, are primary factors contributing to this increased risk of CVDs in postmenopausal women [2–4]. E2 is the most active form of estrogen, mainly produced by the ovaries, and plays a key role in the female reproductive system, as well as other physiological processes via the activation of its receptors, including G protein-coupled estrogen receptor 1(GPER-1), estrogen receptor alpha (ERα), and estrogen receptor beta (ERβ) [5]. ERα and ERβ are mainly genomic receptors that can exert their functions by binding to estrogen response elements in the promoter region of target genes or transcription factor complexes [6]. Also, compared to ERα and ERβ, GPER-1 is more abundant, acts more rapidly, and mostly exerts its function at the membrane and cytosolic levels [6]. Increasing evidence reveals that E2 is cardioprotective through various mechanisms mediated by its receptors during stress [7, 8]. Stress has been identified as a modifiable risk factor for a variety of CVDs [9], and it is an inevitable part of human life [10]. Usually, stress initiated by the demands and complexities of our modern-day lives has a negative impact on overall cardiac health, especially if it is chronic [9, 11]. In a chronic stress state, the continuous activation of the autonomic nervous system characterized by excessive release of catecholamines plays a crucial role in the dysregulation of beta-adrenergic receptors (βARs) in the heart, subsequently resulting in maladaptive inflammatory responses and cardiac remodeling [7, 9, 12].

βARs are 7-transmembrane, G-protein coupled receptors (GPCRs) that regulate the chronotropic and inotropic functions of the heart [13, 14]. Overstimulation and dysregulation of these receptors due to excessive catecholamines activate signaling pathways, which leads to the upregulation of intracellular kinases such as receptor-stimulated p38 mitogen-activated-protein-kinases (p38 MAPKs) and extracellular signal-regulated kinases (ERKs) [9, 15, 16]. Interestingly, these kinases have been implicated in the phosphorylation and activation of 'A Disintegrin and Metalloprotease 17' (ADAM17), a type I transmembrane protein which has gained profound attention in inflammation and tissue remodeling [9, 17]. ADAM17 is ubiquitously expressed in various human body tissues, including the brain, heart, kidneys, and skeletal muscle [9, 18, 19]. Upon activation, this metalloprotease cleaves and triggers several inflammatory mediators and growth factors, including ligands of the epidermal growth factor receptor, cytokines, chemokines, and their receptors [9]. Physiologically, transcriptional factors such as nuclear factor kappa B (NF-κB) and ETS Like-1 (Elk-1) can regulate ADAM17's expression [20]. Additionally, its expression can be influenced by post-transcriptional mechanisms such as the chromatin remodeling of protein BRG1[14].

Several studies have implicated the upregulation of ADAM17's substrates, such as TNFα, in CVDs associated with postmenopausal women [21, 22] and ovariectomized animals [15, 23]. Due to its multifunctionality, developing medications that target ADAM17 has proven to be more difficult than predicted. However, studies have demonstrated that E2 supplementation could reverse and prevent CVDs via the downregulation of inflammatory mediators, including TNFα, a well-known substrate of ADAM17 [15, 23]. And the link between ADAM17 and E2 is not completely elucidated. Thus, it is unclear if E2 can modulate the expressions of ADAM17 during chronic inflammation under stressful conditions. As a result, we explored the effects of E2 deficiency and its replacement on the expressions of ADAM17 during ISO-induced stress in FVB WT female mice and H9c2 cell lines. Additionally, in female C57BL/6 mice, the effects of the E2 receptor GPER-1 on myocardial expressions of ADAM17 were investigated. This study elucidated the mechanism employed by E2 to modulate ADAM17's expression during stress-induced inflammation. Finally, this study demonstrated that E2 or activation of GPER-1 could mitigate stress-induced myocardial inflammation via regulating ADAM17.

## Materials and methods

### Ethics statement

All animal experiments were carried out in strict accordance with the Xuzhou Medical University Guidelines for Animal Experiments and the National Guide for the Care and Use of Laboratory Animals. The Xuzhou Medical University Committee on Animal Experiments reviewed and approved the study protocol (permit number: zx11-12,541).

### Experimental animals and cells

Nine- to ten-week-old female FVB WT and C57BL/6 WT mice supplied by the Laboratory Animal Center of Xuzhou Medical University and C57BL/6 GPER-1 KO and WT mice purchased from Gempharmatech Co., Ltd. (Nanjing, China) were used for in vivo experiments. H9c2 cell lines (GNR5) purchased from the National Collection of Authenticated Cell Cultures (Shanghai, 200,031, China) were used for in vitro experiments. The mice were kept in a room with an average temperature of 25 °C, a 45–55% humidity level, and a 12-h dark–light cycle. The mice were fed and given fresh water regularly. H9c2 Cells (passages 5 to 8) were cultured in DMEM with 10% FBS and 1% penicillin/streptomycin in humidified air containing 5% CO2 at 37 °C.

### Construction of adenovirus encoding ADAM17 siRNA plasmids and transfection

The adenoviral constructs carrying siRNA against a scrambled siRNA (a negative control) and ADAM17 were constructed by Gene Pharma (Shanghai, 1054 SUN ET AL. China). The targeted sequences for ADAM17 are listed below in Table 1. The H9c2 cells were cultured in a 60 mm dish under normal conditions and then transfected with adenovirus-mediated siRNA against ADAM17 and scrambled siRNA using lipofectamine 2000 (Sigma) for 6 h according to the manufacturer's instructions. The transfected cells were visualized for GFP expression and treated for experiments.

**Table 1:** ADAM17'S siRNA sequences

No	Targets	Forward	Reverse
1	Negative control	5′-UUCUCCGAACGUGUCACGUTT-3	5′-ACGUGACACGUUCGGAGAATT-3’
2	Negative control FAM	5′-UUCUCCGAACGUGUCACGUTT-3	5′-ACGUGACACGUUCGGAGAATT-3’
3	Rat Actb-324	5′CUCUGAACCCUAAGGCCAATT-3	5′-UUGGCCUUAGGGUUCAGAGGG-3’
4	Adam17-Rat-326	CCUGCGACUUGAGAAGCUUTT	AAGCUUCUCAAGUCGCAGGTT
5	Adam17-Rat-1775	GCAGUGCAGUGAUAGGAAUTT	AUUCCUAUCACUGCACUGCTT
6	Adam17-Rat-1193	GGAGCAAUUUAGCCUUGAUTT	AUCAAGGCUAAAUUGCUCCTT
7	Adam17-Rat-1196	GCAAUUUAGCCUUGAUAUATT	UAUAUCAAGGCUAAAUUGCTT

### Animal model

**Fig. 1:**
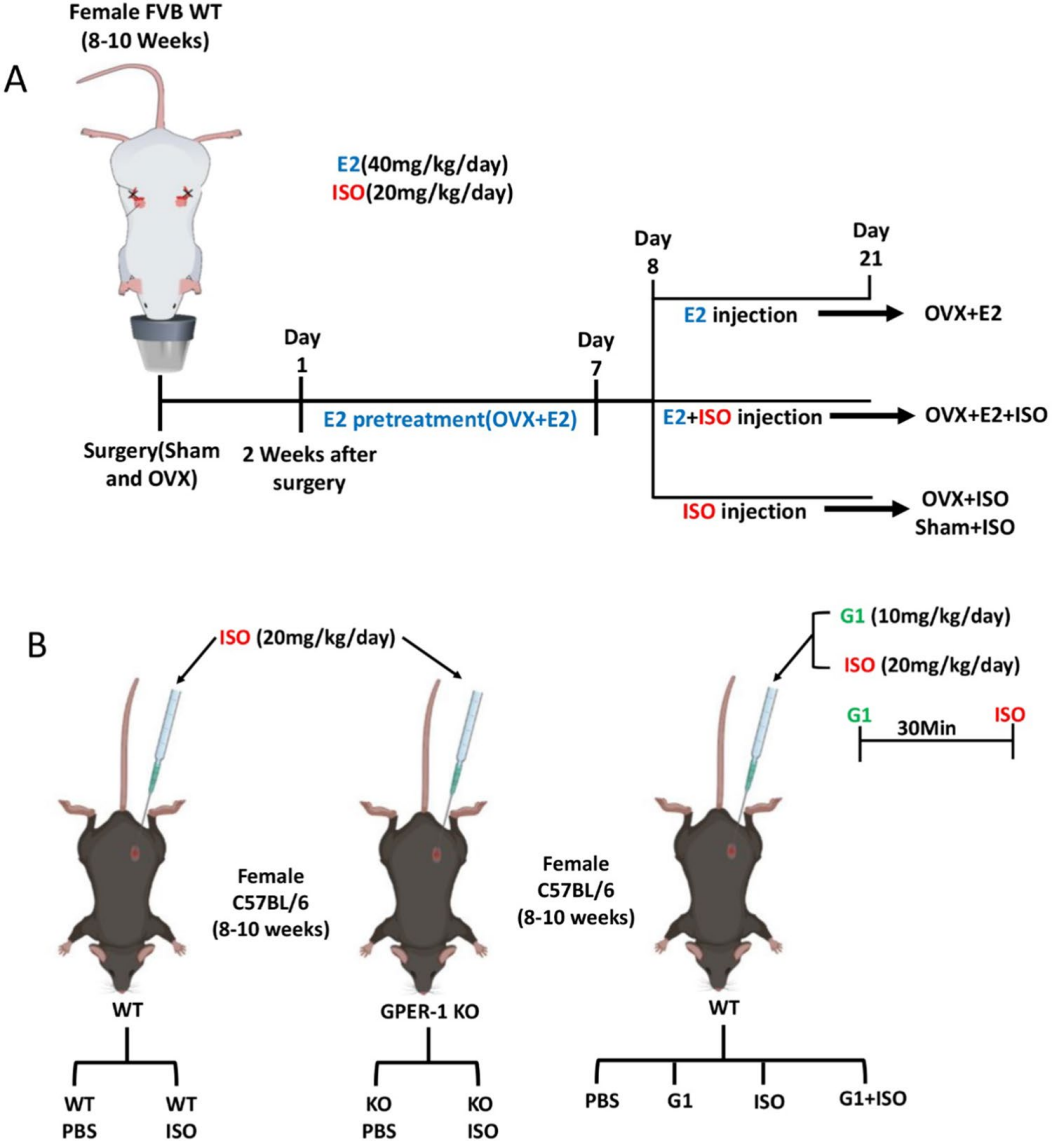
Graphical representation of experimental timeline for in vivo studies **A** Graphical outline of in vivo experiments in female FVB WT mice. **B** Graphical outline of in vivo experiments in female C57BL/6 WT and KO mice

Female FVB WT mice were sham-operated and ovariectomized (OVX), then divided into six groups based on the treatment (*n* = 4–8 mice per group depending on the experimental setting). The mice were anesthetized prior to the sham operation and ovariectomy. Sham and OVX surgery was performed as previously described [24]. Corneal reflex disappearance, reduction in pain sensation, and decreased muscle tension were signs of successful anesthesia. Following anesthetization, mice were placed in the prone position and had their caudal half-back disinfected with povidone-iodine. A subcutaneous incision was made at the right side of the spine, separating the muscle layer and opening the peritoneum. The right ovary (red cauliflower-like milky white fat) was located in the abdominal cavity. The right ovarian artery was ligated, and the right ovary was removed. The fallopian tube and surrounding fat were dressed with antibiotics (penicillin) and pushed back into the abdominal cavity. Peritoneum and skin suturing were then performed. The same procedure was carried out on the left ovary. In the Sham group, the ovaries were visualized by incisions into the abdominal cavity, followed by peritoneum and skin suturing. All surgeries were performed aseptically and in a sterile environment. After two weeks, the mice were subcutaneously administered with ISO (20 mg/kg/day) (Sigma, 16,504) and E2 (40 mg/kg/day) (Sigma, 50–28-2) for two weeks and three weeks, respectively (Fig. 1) as previously demonstrated [25, 26]. Also, female C57BL/6 WT and GPER-1 KO and WT mice were categorized into groups (n = 4–5 mice per group) based on the subcutaneous administration of PBS (vehicle), ISO (20 mg/kg/day) and G1(10 mg/kg/day) for two weeks (Fig. 1).

### Cell model

Following ADAM17 siRNA transinfection in H9c2 cells, ADAM17-siRNA and ADAM17^+/+^ cells were treated with 10 µM of ISO, 1 nM of E2, 11 nM of GPER-1 agonist (G1)(Cayman; 10,008,933), 20 nM of GPER-1 antagonist (G15) (Cayman; 14,673), and 20 nM of PI3K inhibitor (LY294002) (Sigma; 24,278,828) for 24 h. The specific concentrations for the in vitro experiments were selected based on the manufacturer's recommendations and published literature[7, 27, 28].

### RNA extraction and real-time qPCR

TRIzol (Life technologies; 15,596,026) was used to extract total RNA from the ventricular myocardium (*n* = 4–6 hearts per treatment group) and treated H9c2 cells (*n* ≥ 2 ∗ 10^6^ cells per treatment group). Synthetization of cDNA from 1 g of RNA was performed using a reverse transcription kit based on the manufacturer's instructions (Toyobo; FSQ107). The semiquantitative RT-PCR was employed to amplify the obtained cDNAs using SYBR Green Master Mix (Vazyme; Q111-02). The primer sequences are enumerated in Table 2. The relative quantification of gene expression was calculated using the 2 − ΔΔCt technique.

**Table 2:** Primer sequences for real-time qPCR

No	Primers	Forward	Reverse
1	ADAM17	GAAAGAAGAAAGCGAGTACAGC	CATCATCTCCTATGTGGGCTAG
2	TNFα	CTTGTTGCCTCCTCTTTTGCTTA	CTTTATTTCTCTCAATGACCCGTA
3	IL-1β	TGTGTTTTCCTCCTTGCCTCTGAT	TGCTGCCTAATGTCCCCTTGAAT
4	IL-10	GGAAGACAATAACTGCACCCACT	CAACCCAAGTAACCCTTAAAGTCC

### Tissue sectioning and immunohistochemistry

Whole hearts (*n* = 4–6 mice per treatment group) were harvested and fixed with isopentane pre-cold in liquid nitrogen for 10 s. The rapidly frozen hearts were then sectioned longitudinally at a thickness of 5 μm. Following rehydration in deionized water, tissue sections were rinsed three times in PBS (3 min). Non-specific antibody binding sites were blocked using H_2_O_2_ for 10 min, followed by 3% BSA for 30 min. The tissue sections were then incubated overnight at a constant temperature of 4 °C with the appropriate primary antibodies: CD68 (Abcam; Ab955), CD86(Abcam; ab53004), and CD206(Abcam; ab8918). The next day, tissues were incubated with biotinylated goat anti-rabbit IgG and streptavidin peroxidase for 20 and 30 min, respectively. Subsequently, DAB (ORIGENE; ZLI-9018) staining and hematoxylin counterstaining were performed, followed by dehydration in ascending series of ethanol. The tissue slides were then dried in xylene and mounted for microscopy. Tissue slides were imaged at a magnification of 400 × and analyzed using ImageJ (1.52a version; National Institute of Health, United States).

### Enzyme-linked immunosorbent assay (ELISA)

The concentrations of proinflammatory cytokines (TNFα and IL-1β), anti-inflammatory cytokine (IL-10), eNOS, and cardiac injury markers (ANP, BNP, and cTnI) were determined using sera and cell culture media supernatants from in vivo and in vitro models, respectively. Mouse TNFα kits (Proteintech; KE10002), rat TNFα kits ( Proteintech; KP00000977), mouse IL-1β kits (Proteintech; KP00000813), rat IL-1β kits (Jianglai Biota; JL20884), mouse IL-10 kits ( Proteintech; KE10008), mouse ANP kits (Jianglai Biota; JL20612), mouse BNP kits (Jianglai Biota; JL20884), mouse cTnI kits (Signalway; EK1821), rat ANP kits (Jianglai Biota; JL21352), rat BNP kits (Jianglai Biota; JL11495), rat cTnI kits (Signalway; EK1819), rat eNOS kits (Jianglai Biota; JL21190) ELISA was performed in triplicates according to the instructions of the manufacturer.

### Western blot

Harvested heart ventricles (*n* = 4–6 mice per treatment group) and treated H9c2 cells (*n* ≥ 2 ∗ 10^6^ cells per treatment group) were homogenized in a lysis buffer containing phosphatase and proteinase cocktail inhibitor in a 100:1:1 ratio, respectively. Lysates' concentrations were normalized and denatured at 100 °C for 10 min. Equal amounts (20 μl) of protein extracts were run on a sodium dodecyl sulfate (SDS)-polyacrylamide gel electrophoresis and electro-transferred onto a nitrocellulose membrane (Millipore, Darmstadt, Germany). The transferred protein bands were blocked in Tris-buffered saline (TBS)-0.1% Tween 20 solution (TBST) with 3% bovine serum albumin (BSA) under room temperature for an hour. The membrane was then incubated overnight at a constant temperature of 4 °C with the following primary antibodies: ADAM17(1:1000, Abcam), AKT(1:1000, Cell Signalling Technology), pAKT(1:1000, Cell Signalling Technology), β1AR(1:1000, Abcam), β2AR(1:1000, Abcam), β-actin (1:10,000, Proteintech), ERK1/2(1:1000, Cell Signalling Technology), pERK1/2(1:1000, Cell Signalling Technology), GPER-1(1:500, Abcam), GAPDH (1:10,000, Abcam), Gαi(1:1000, NewEast Bioscience), Gαs(1:1000, NewEast Bioscience), PI3K(1:1000, Cell Signalling Technology), pPI3K(1:1000, Cell Signalling Technology), P38(1:1000, Cell Signalling Technology), pP38(1:1000, Cell Signalling Technology), NF-κB(1:1000, Abmart), pNF-κB(1:1000, Abmart). The membranes were then incubated with corresponding secondary antibodies (1:4000, Ptoteintech n, Beijing, China) at room temperature for 2 h. Each membrane was subsequently treated with appropriate secondary antibodies conjugated to horseradish peroxidase after washing four times (5 min each) with Tris-buffered saline (TBS)-0.1% Tween 20 solution. The bands were then visualized using enhanced chemiluminescence (Millipore Darmstadt, Germany). Immunoblots were repeated three times and normalized using their appropriate loading controls.

### Immunofluorescence staining

Formalin (4%) was used to fix cultured and treated H9c2 cells (n ≥ 2 ∗ 10^6^ cells per treatment group). Triton x (0.1%)(VICMED; 9005–64-5) was used to permeate cells for 15 min. Binding sites of non-specific antibodies were blocked for 1 h in PBS with 1% BSA. H9c2 cells were then treated and incubated overnight at a constant temperature of 4 °C with ADAM17 primary antibody (Abcam; ab2051), then rinsed with PBS, and probed with R-PE-conjugated secondary antibody (Proteintech; SA00008-2) for 1 h under room temperature. Subsequently, the cells were washed with PBS and conditioned with 0.5% BSA in Hanks' balanced salt solution. The cytoplasmic membranes of cells were then stained for 30 min at 4 °C with WGA (Thermo Fisher Scientific; W11261). DAPI (Beyotime; C1005) nuclei staining was performed, followed by imaging and assessment of membrane, cytoplasmic, and nucleic expressions ratios of ADAM17 with ImageJ (*n* = 25–35 cells per disk).

### Statistical analysis

Sample sizes were determined by Power analysis, and all data were analyzed by GraphPad Prism (Prism Version 8.0.2). An unpaired t-test was utilized for comparing two groups, while a one-way ANOVA was employed for comparing three or more groups. A two-way ANOVA was used for statistical analysis in the case of grouped data. Post hoc analyses were performed using Tukey’s multiple comparison test. All results were presented as mean ± SEM, and statistical significance was assigned as *P* < 0.05. All experiments are represented by multiple biological replicates.

## Result

### E2 decreased cardiac injury and inflammation in FVB Mice during ISO-induced stress.

**Fig. 2:**
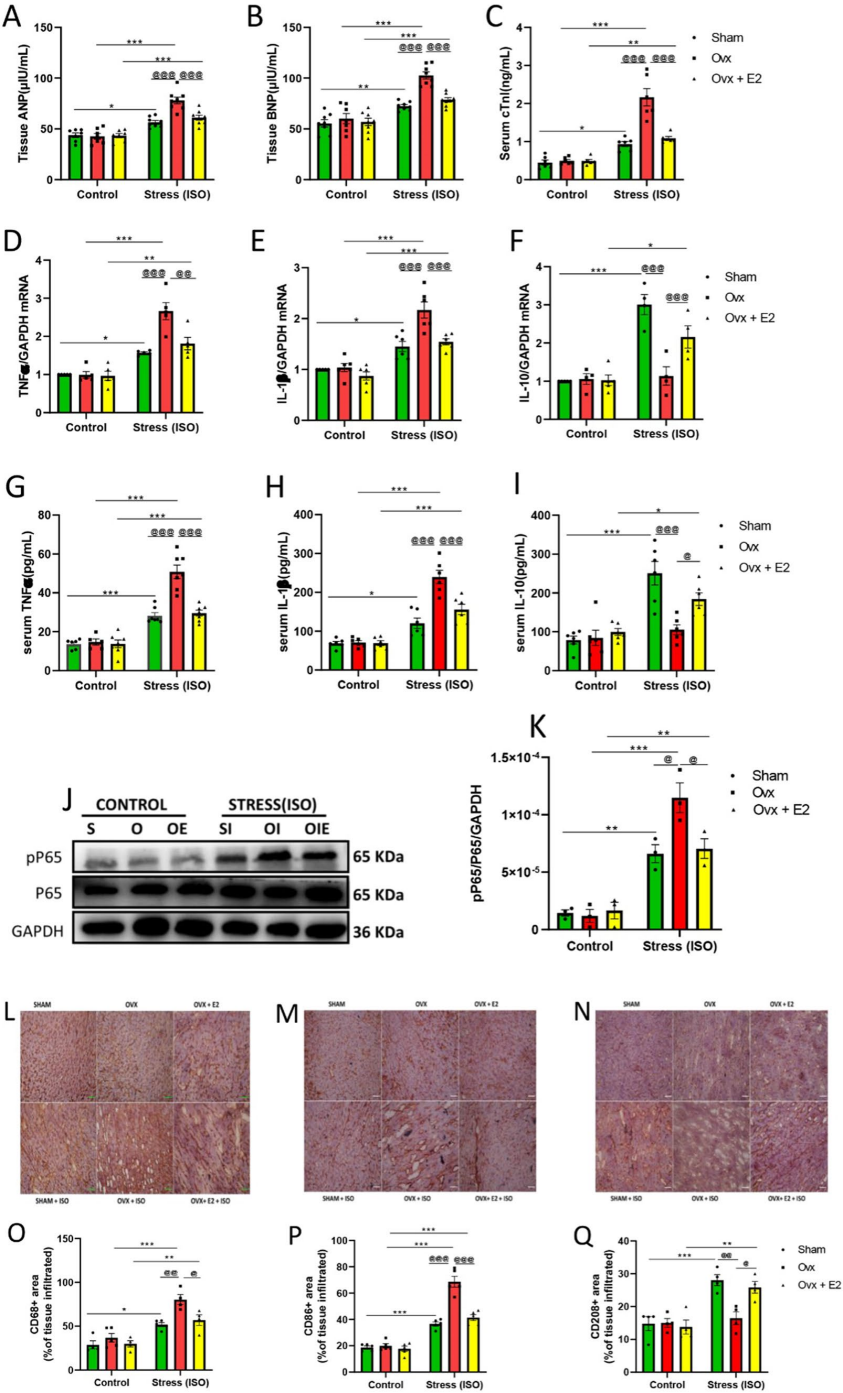
Estradiol (E2) reduces cardiac injury and inflammation in FVB female mice during ISO stress. **A**, **B**, **C** Effect of E2 on the concentrations of cardiac injury markers in cardiac tissues (*n* = 6–8). **D**, **E**, **F** Effect of E2 on the RNA concentrations of inflammatory cytokines (*n* = 4–5). **G**, **H**, **I** Effect of E2 on the serum concentrations of inflammatory cytokines (*n* = 6). **J**, **K** Effect of E2 on cardiac protein expression of NF-κB (p65) (*n* = 3). **L**, **M**, **N** Picture representation of macrophage infiltration in cardiac tissues (*n* = 4–5), (scale bar = 50 μm). **O**, **P**, **Q** Graphical representation of macrophage infiltration in cardiac tissues (*n* = 4–5). Two-way ANOVA results are presented as mean ± SEM, *Comparison between control and its stressed group, ^@^Comparison among stressed groups (*p < 0.05 or ^@^*p* < 0.05.) (**P < 0.01 or ^@@^*P* < 0.01), (***P < 0.001 or ^@@@^*P* < 0.001). Sham(S), Ovx(O), Ovx + E2(OE), Sham + ISO(SI), Ovx + ISO(OI), Ovx + ISO + E2(OIE)

Cardiac injury markers, including atrial natriuretic peptide (ANP), brain natriuretic peptide (BNP), and cardiac troponin I(cTnI), were measured using ELISA (Fig. 2A–C). qPCR and ELISA were employed to determine the RNA and serum levels of proinflammatory (TNFα, IL-1β) and anti-inflammatory (IL-10) cytokines (Fig. 2D–I), and finally, western blot was used to measure NF-κB (P65) protein expression (Fig. 2J, K). Myocardial inflammation was assessed by performing immunohistochemistry (IHC) to ascertain tissue infiltration of macrophages (CD68 + , CD86 + , CD206 +) (Fig. 2L–Q). Significant elevation of myocardial ANP and BNP, along with a notable rise in serum cTnI, were associated with the OVX + ISO group. In addition, elevated RNA and serum levels of proinflammatory cytokines (TNFα, IL-1β) were observed in the OVX + ISO group compared with its control group. A significant increase in macrophage infiltration (CD68 +) was seen in the OVX + ISO group, with more proinflammatory macrophages (CD86 +) and fewer reparative macrophages (CD206 +). The endogenous E2 (in the SHAM + ISO) and supplementation of exogenous E2 (in the OVX + E2 + ISO) mediated the switch of proinflammatory to reparative macrophages, decreasing the total macrophage infiltration in cardiac tissues. Similarly, E2 significantly decreased the RNA and serum levels of proinflammatory cytokines (TNFα, IL-1β) and cardiac injury makers (ANP, BNP, cTnI), with a remarkable increase in anti-inflammatory cytokine, IL-10 (Fig. 2).

### E2 downregulates the expressions of ADAM17 in female FVB Mice during ISO-induced chronic stress

**Fig. 3:**
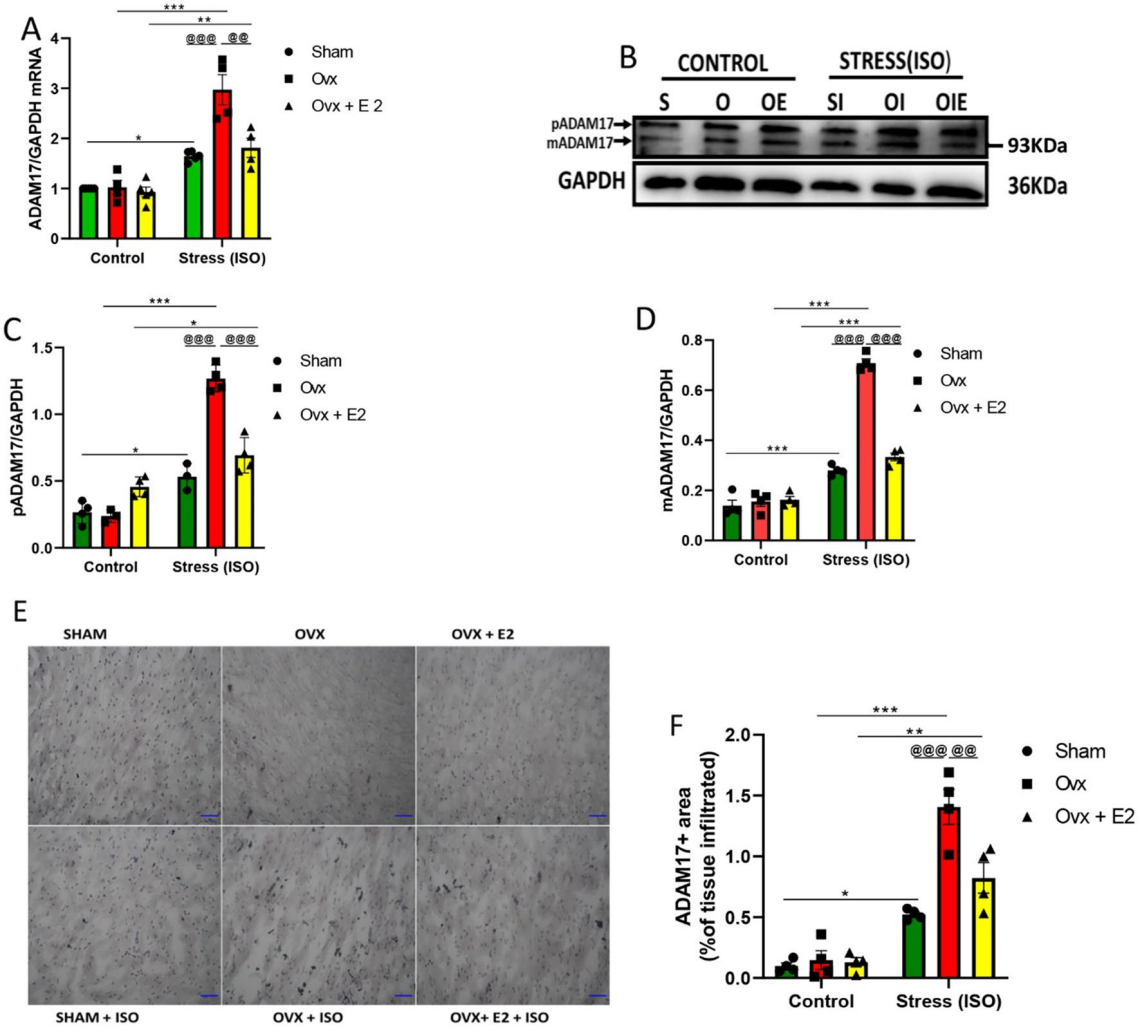
Estradiol (E2) downregulates myocardial ADAM17 during stress-induced inflammation in FVB female mice. **A** Effect of E2 on myocardial RNA concentration during ISO-induced stress (*n* = 4–5). **B**, **C**, **D** Effect of E2 on the cytoplasmic(pADAM17) and membrane content of ADAM17(mADAM17) during ISO-induced stress (*n* = 3–4). **E**, **F** Effect of E2 on the myocardial infiltration of ADAM17 during ISO-induced stress, (scale bar = 50 μm), (*n* = 4). Two-way ANOVA, results are presented as mean ± SEM, *Comparison between control and its stressed group, ^@^Comparison among stressed groups (*p < 0.05 or ^@^*p* < 0.05.) (**P < 0.01 or ^@@^*P* < 0.01), (***P < 0.001 or ^@@@^*P* < 0.001). Sham(S), Ovx(O), Ovx + E2(OE), Sham + ISO(SI), Ovx + ISO(OI), Ovx + ISO + E2(OIE)

The myocardial expression of ADAM17 was ascertained using qPCR, western blot, and IHC to measure the RNA levels, protein expressions, and tissue infiltration of ADAM17, respectively (Fig. 3A–F). It was observed that significant upregulation of myocardial ADAM17, along with remarkable tissue infiltration, was associated with the OVX + ISO group during stress. Nevertheless, the endogenous E2 (in the SHAM + ISO) and supplementation of exogenous E2 (in the OVX + E2 + ISO) significantly decreased its levels and tissue infiltration. Furthermore, the western blot analysis revealed a significant decrease in myocardial expression of pro-ADAM17(pADAM17) and membrane content of ADAM17(mADAM17) in SHAM + ISO and OVX + E2 + ISO groups compared to the OVX + ISO group.

### Activation of GPER-1 downregulates the expression of myocardial mADAM17 during ISO-induced stress in C57BL/6 female mice

**Fig. 4:**
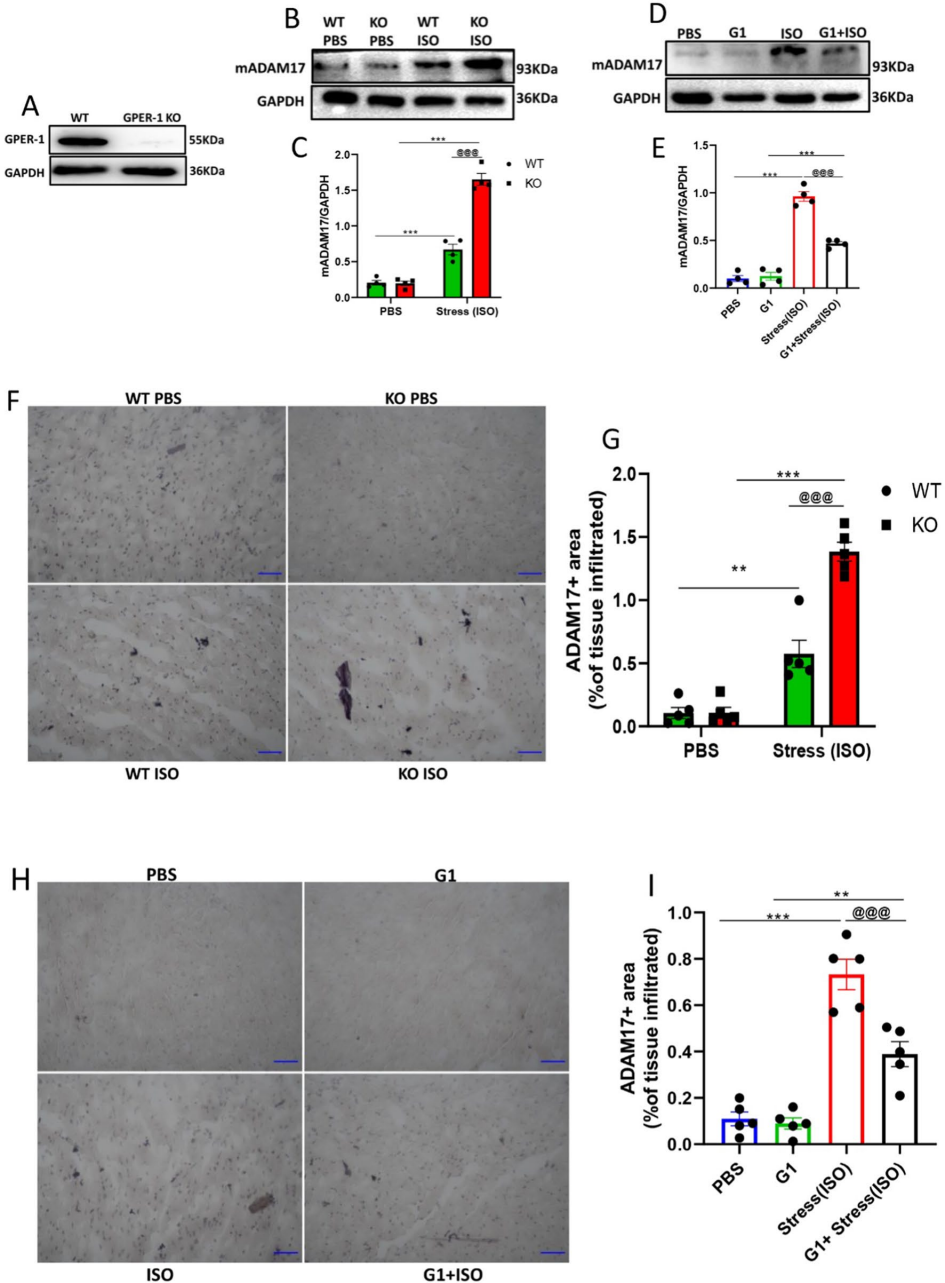
Activation of GPER-1 downregulates myocardial mADAM17 during ISO-induced stress in C57BL/6 female mice. **A** Protein expression of GPER-1 in the cardiac tissues of WT and KO. **B**, **C** Effect of GPER-1 knockout on the membrane content of ADAM17 in cardiac tissues during ISO-induced stress (*n* = 4). **D**, **E** Effect of G1 administration on the membrane content of ADAM17in cardiac tissues during ISO-induced stress (*n* = 4). **F**, **G** Effect of GPER-1 knockout on the myocardial infiltration of ADAM17 during ISO-induced stress, (scale bar = 50 μm), (*n* = 5). **H**, **I** Effect of G1 administration on the myocardial infiltration of ADAM17 during ISO-induced stress, (scale bar = 50 μm), (*n* = 5). Two-way ANOVA and one-way ANOVA results are presented as mean ± SEM, *Comparison between control and its stressed group, ^@^Comparison among stressed groups (*p < 0.05 or ^@^*p* < 0.05.) (**P < 0.01 or ^@@^*P* < 0.01), (***P < 0.001 or ^@@@^*P* < 0.001)

To investigate the effects of GPER-1 on the expression of myocardial ADAM17 during stress, female C57BL/6 KO mice were employed (Fig. 4A–C, F, G). Additionally, 10 mg/kg/day of G1 was used for the activation of GPER-1 to ensure its full activity at a realistic, pharmacologically achievable dose as previously described [29] (Fig. 4D, E, H, I). During stress in female C57BL/6 mice, the expression of ADAM17 in the myocardia was assessed using western blot and IHC to ascertain its protein expression and tissue infiltration, respectively. Compared to the WT + ISO and WT + PBS groups, the GPER-1 KO + ISO group showed a significant upregulation of myocardial mADAM17 and remarkable tissue infiltration. Also, administration of G1 (in the G1 + ISO) significantly decreased the expression of mADAM17 and its tissue infiltration in the myocardia during stress (Fig. 4).

### E2 downregulates the expression of mADAM17 via the activation of the GPER-1 signaling pathway in FVB female mice and H9c2 cell lines

**Fig. 5:**
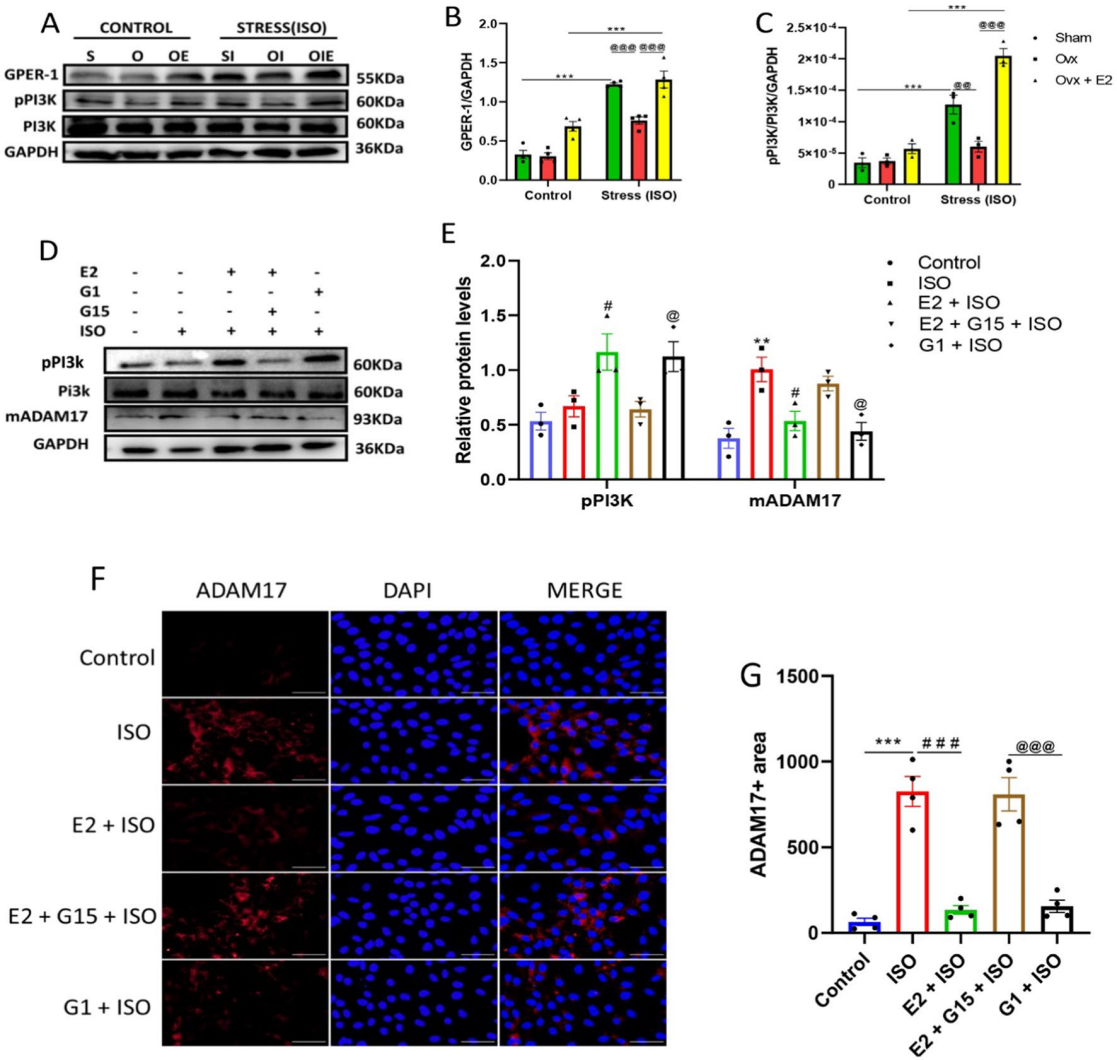
Estradiol (E2) downregulates myocardial mADAM17 via GPER-1 during stress-induced inflammation in FVB female mice and H9c2 cell lines. **A**, **B**, **C** Effect of E2 on the myocardial protein expression of GPER-1 and pPI3K in cardiac tissues of FVB mice under ISO-induced stress (*n* = 3–4). **D**, **E** Western blot analysis displaying the effect of E2 via GPER-1 and its down signaling molecule, pPI3K, on the protein expression of mADAM17 in H9c2 cells during ISO-induced stress (Triplicate per treatment group). **F**, **G** Immunofluorescence analysis showing the effect of E2 via GPER-1 on mADAM17’s expression during ISO-induced stress; nuclei (DAPI), membrane (WGA), (*n* = 25–35 per disk, quadruplicate per treatment group), (scale bar = 50 μm). Two-way ANOVA for in vivo analysis, results are presented as mean ± SEM, *Comparison between control and its stressed group, ^@^Comparison among stressed groups. One-way ANOVA for in vitro analysis, results are presented as mean ± SEM, *Comparison between control and ISO groups, ^#^Comparison between ISO and E2 groups, ^@^Comparison between G15 and G1 groups. (*p < 0.05 or ^#^ *p* < 0.05 or ^@^*p* < 0.05.) (**P < 0.01 or ^##^ *P* < 0.01 or ^@@^*P* < 0.01), (***P < 0.001 or ^###^*P* < 0.001 or ^@@@^*P* < 0.001). Sham(S), Ovx(O), Ovx + E2(OE), Sham + ISO(SI), Ovx + ISO(OI), Ovx + ISO + E2(OIE)

During stress in FVB female mice, the activation of GPER-1 signaling was determined by assessing the protein levels of GPER-1 and its down signaling protein, phosphorylated PI3K (pPI3K), using western blots (Fig. 5A−C). Confirmation was done by carrying out in vitro experiments on H9c2 cell lines; thus, following the treatment of H9c2 cells with E2 and G15(GPER-1 inhibitor), and G1(GPER-1 agonist) under ISO-induced stress conditions, the protein expressions of GPER-1 and pPI3K were determined by western blot, along with the assessment of mADAM17 by western blot and immunofluorescence method (Fig. 5A–G). In vivo experiments showed significant upregulation of GPER-1 and PI3K in SHAM + ISO and OVX + E2 + ISO groups compared with the OVX + ISO group. The in vivo experiments confirmed these findings by showing a remarkable decrease in pPI3K expression along with a significant increase in mADAM17's protein expression after the inhibition of GPER-1 with G15 in the presence of E2 and ISO. In addition, the activation of GPER-1 with G1 in the presence of ISO significantly upregulated pPI3K levels and considerably decreased the membrane content of ADAM17 (Fig. 5).

### E2 downregulates the expression of mADAM17 via the upregulation of the PI3K/AKT/eNOS signaling pathway in FVB female mice and H9c2 cell lines

**Fig. 6:**
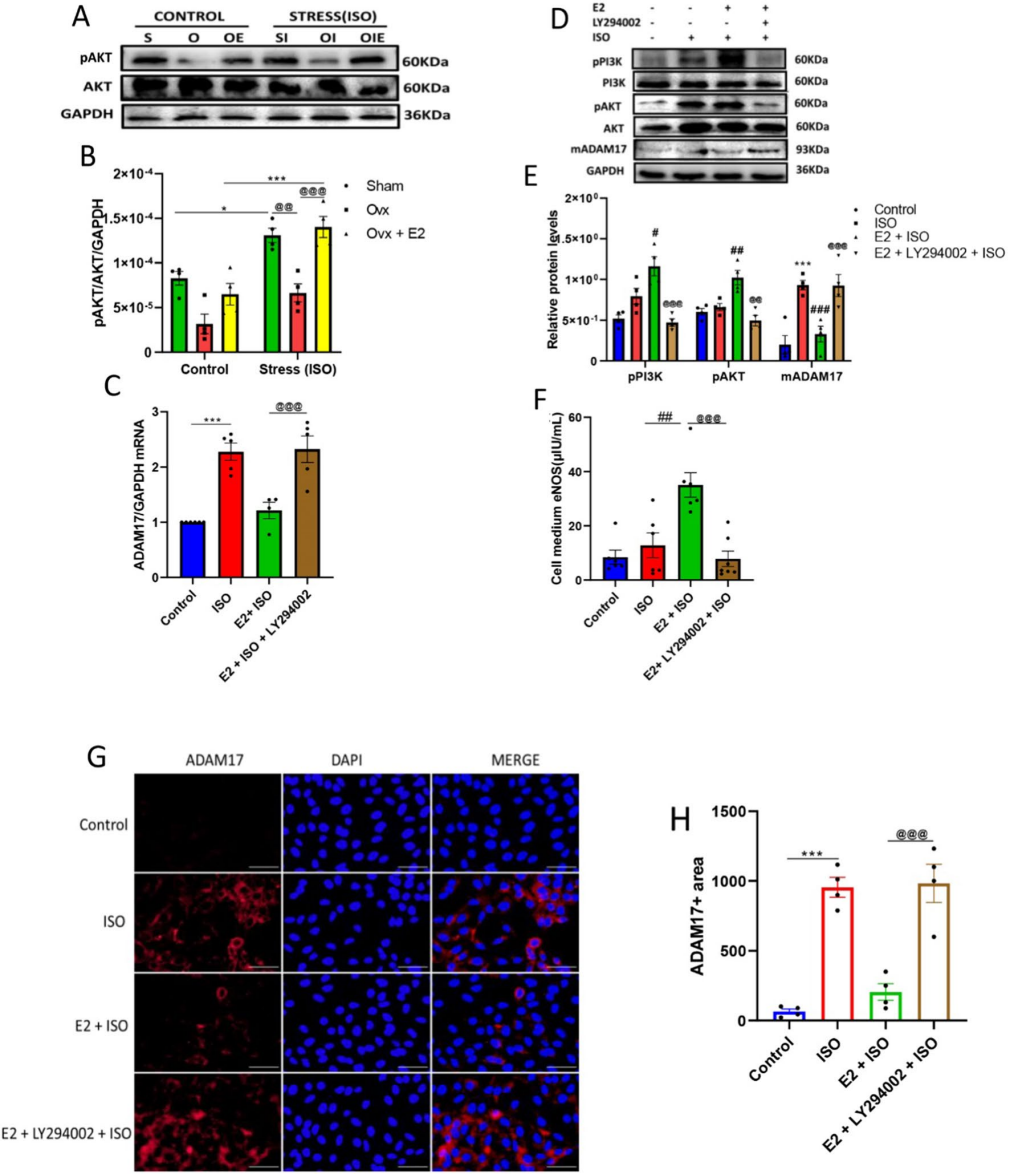
Estradiol (E2) downregulates myocardial mADAM17 via the upregulation of the PI3K/AKT/eNOS pathway during stress-induced inflammation in FVB female mice and H9c2 cell lines. **A**, **B** Effect of E2 on the protein expression of pAKT in cardiac tissues of FVB mice under ISO-induced stress (*n* = 3–4). **C** Effect of E2 via PI3K on the myocardial RNA levels of ADAM17 during ISO-induced stress;(Triplicate per treatment group). **D**, **E** Western blot analysis displaying the effect of E2 via PI3K and its down signaling molecule, pAKT on the protein expression of mADAM17 in H9c2 cells under ISO stress condition (Triplicate per treatment group). **F** ELISA analysis showing the effect of E2 via PI3K on culture media levels of eNOS under ISO stress condition (Triplicate per treatment group). **G**, **H** Immunofluorescence analysis displaying the effect of E2 via PI3K on mADAM17’s expression under ISO stress condition; nuclei (DAPI), membrane (WGA), (*n* = 25–35 per disk, quadruplicate per treatment group), (scale bar = 50 μm). Two-way ANOVA for in vivo analysis, results are presented as mean ± SEM, *Comparison between control and its stressed group, ^@^Comparison among stressed groups. One-way ANOVA for in vitro analysis, results are presented as mean ± SEM, *Comparison between ISO and control groups, ^#^Comparison between ISO and E2 groups, ^@^Comparison between E2 and LY294002 groups (*p < 0.05 or ^#^ *p* < 0.05 or ^@^*p* < 0.05.) (**P < 0.01 or ^##^*P* < 0.01 or ^@@^*P* < 0.01), (***P < 0.001 or ^###^*P* < 0.001 or ^@@@^*P* < 0.001). Sham(S), Ovx(O), Ovx + E2(OE), Sham + ISO(SI), Ovx + ISO(OI), Ovx + ISO + E2(OIE)

During ISO-induced stress in FVB female mice, the significant upregulation of pPI3K and its down signaling molecules, pAKT and eNOS, were observed in SHAM + ISO and OVX + E2 + ISO groups compared to the OVX + ISO group (Fig. 6A, B). Validation of these findings was done by performing in vitro experiments on H9c2 cell lines; thus, treatment of H9c2 cells with E2 and PI3K inhibitor (LY294002) in the presence of ISO was carried out, followed by the assessment of pPI3K, pAKT, eNOS, and ADAM17 protein expressions (Fig. 6C–H). qPCR analysis revealed a notably reduced RNA level of ADAM17 in E2 + ISO compared with ISO and E2 + LY294002 + ISO groups. Western blots analysis revealed a considerable increase in pPI3K and pAKT, along with a significant decrease in mADAM17's expression in the E2 + ISO group compared to the ISO group. Similarly, ELISA analysis showed a notable elevation of eNOS levels in the culture media of the E2 + ISO group compared to the ISO group. On the flip side, inhibition of PI3K reversed the cardioprotective effect of E2 by significantly decreasing pAKT and media levels of eNOS in the E2 + LY294002 + ISO group compared to the E2 + ISO group. Furthermore, immunofluorescence analysis supported these findings, which showed a considerable increase in mADAM17 expression after the blockage of PI3K in the presence of E2 under ISO stress conditions (Fig. 6).

### E2 via PI3K signaling downregulated mADAM17 expressions through perinuclear localization.

**Fig. 7:**
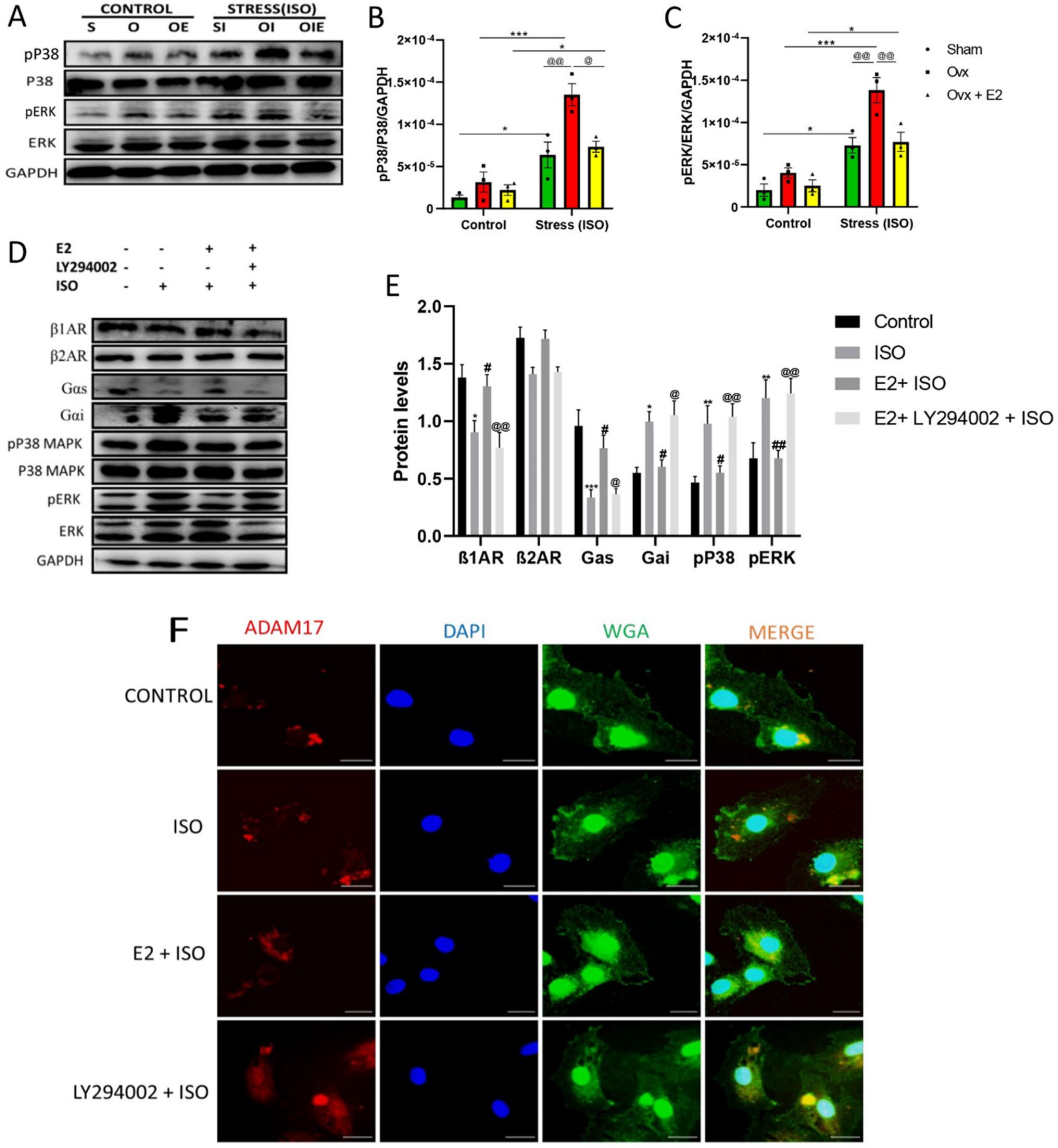
Estradiol (E2) via PI3K downregulates mADAM17 through peri-nuclear localization in FVB female mice and H9c2 cell lines. **A**, **B**, **C** Effect of E2 on the protein expression of phosphorylated ERKs and p38MAPKs in cardiac tissues of FVB mice under ISO-induced stress (*n* = 3–4). **D**, **E** Western blot analysis displaying the effect of E2 via PI3K on the protein expressions of β1AR, β2AR, Gαs, Gαi, phosphorylated ERKs and p38MAPKs in H9c2 cells under ISO stress condition (Triplicate per treatment group). **F** Immunofluorescence analysis displaying the effect of E2 via PI3K on mADAM17’s localization during ISO-induced stress; nuclei (DAPI), membrane (WGA), (Triplicate per treatment group), (scale bar = 50 μm). Two-way ANOVA for in vivo analysis, results are presented as mean ± SEM, *Comparison between control and its stressed group, ^@^Comparison among stressed groups. One-way ANOVA for in vitro analysis, results are presented as mean ± SEM, *Comparison between ISO and control groups, ^#^Comparison between ISO and E2 groups, ^@^Comparison between E2 and LY294002 groups. (*p < 0.05 or ^#^*p* < 0.05 or ^@^*p* < 0.05.) (**P < 0.01 or ^##^*P* < 0.01 or ^@@^*P* < 0.01), (***P < 0.001 or ^###^*P* < 0.001 or ^@@@^*P* < 0.001). Sham(S), Ovx(O), Ovx + E2(OE), Sham + ISO(SI), Ovx + ISO(OI), Ovx + ISO + E2(OIE)

During ISO-induced stress in FVB female mice, notable downregulation of phosphorylated ERKs and p38MAPKs was observed in the SHAM + ISO and OVX + E2 + ISO groups compared to the OVX + ISO group (Fig. 7A–C). Confirmation of these findings was done by performing in vitro experiments on H9c2 cell lines; thus, treatment of H9c2 with E2 and PI3K inhibitor (LY2940002) in the presence of ISO was carried out, followed by the assessment of β1AR, β2AR, Gαs, Gαi, phosphorylated ERKs and p38MAPKs along with immunofluorescence assessment of ADAM17 protein localization (Fig. 7D–F). Western blots analysis showed a remarkable increase in the protein expressions of β1AR along with a significant increase in Gαs protein expression and a notable decrease in Gαi protein expression in the E2 + ISO group compared to the ISO group. Although elevated protein expression of β2AR was observed in the E2 + ISO group compared to the ISO and E2 + LY294002 + ISO groups, the difference was not statistically significant. The inhibition of PI3K with LY294002 reversed the E2 action by significantly downregulating the protein expressions of β1AR along with elevated phosphorylated ERKs and p38MAPKs. Localization assessments revealed increased peri-nuclear localization in control and E2 + ISO groups compared to the ISO and E2 + LY294002 + ISO groups.

### The downregulation of ADAM17's expression by E2 attenuated ISO-induced injury and inflammation in H9c2 cells.

**Fig. 8:**
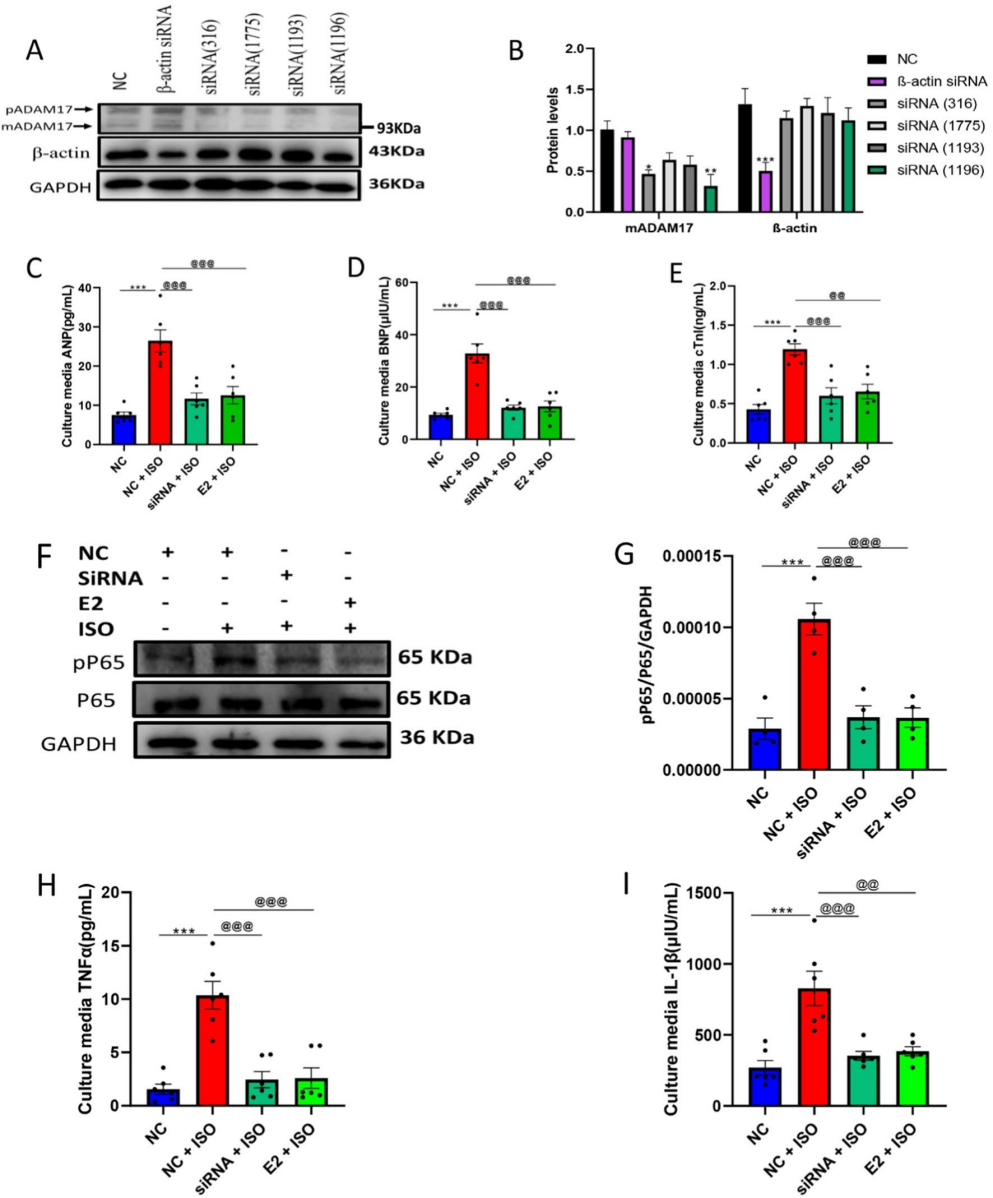
The downregulation of ADAM17 by estradiol(E2) attenuates ISO-induced cardiac injury and inflammation in H9c2 cell lines. **A**, **B** Western blot analysis of ADAM17 siRNA targets in H9c2 cells (Triplicate per treatment group). **C**, **D**, **E** ELISA analysis of ANP, BNP and cTnI levels in the culture media of treated H9c2 cells during ISO-induced stress (quadruplicate per treatment group). **F**, **G** Western blot analysis displaying the effect of E2 via ADAM17 on the protein expression of NF-κB (P65) in H9c2 cells under ISO stress condition (Triplicate per treatment group). **H**, **I** ELISA analysis of TNFα and IL-1β levels in the culture media of treated H9c2 cells during ISO-induced stress (quadruplicate per treatment group). One-way ANOVA results are presented as mean ± SEM, *Comparison between treated cells and their control groups, ^@^Comparison between ISO groups, (*p < 0.05 or ^#^ *p* < 0.05 or ^@^*p* < 0.05.) (**P < 0.01 or ^##^*P* < 0.01 or ^@@^*P* < 0.01), (***P < 0.001 or ^###^*P* < 0.001 or ^@@@^*P* < 0.001). Negative control (NC)

Silencing of the ADAM17 gene by siRNA was carried out on H9c2 cells and confirmed using western blot analysis (Fig. 8A, B). The best siRNA was selected and used for further experiments to ascertain the effect of ADAM17 on cardiac inflammation; thus, ADAM17^+/+^ cells with E2 treatment and ADAM17-siRNA cells were placed under the same ISO condition, followed by the culture media assessment of cardiac injury markers (ANP, BNP, cTnI), proinflammatory cytokines (TNFα and IL-1β), and protein assessment of NF-κB (P65) (Fig. 8C–I). Western blot analysis showed a significant upregulation of NF-κB (P65) in the siRNA   NC + ISO group compared to the siRNA NC group. Similarly, ELISA assessment revealed a remarkable increase in culture media levels of proinflammatory cytokines (TNFα, IL-1β) and cardiac injury markers (ANP, BNP, cTnI) in the siRNA  NC + ISO group compared to the siRNA NC group. However, the silencing of ADAM17 (in siRNA + ISO) and the supplementation of E2 (in E2 + ISO) considerably downregulated the protein expressions of NF-κB (P65) along with a reduction in the culture media levels of proinflammatory cytokines (TNFα and IL-1β), and cardiac injury markers (ANP, BNP, cTnI) compared to the siRNA NC + ISO group (Fig. 8).

## Discussion

Stress-induced CVDs characterized by inflammation are one of the primary causes of morbidity and mortality in postmenopausal women globally [1]. TNFα, which plays a central role in inflammatory diseases, is primarily converted to its active form by ADAM17, a metalloproteinase that specializes in releasing membrane-tethered proteins. This protease has garnered considerable attention as its cleavage abilities appear to be linked to key modern pathologies like cancer, inflammatory, and vascular diseases [30]. Since its discovery as the tumor necrosis factor convertase, it has been a prominent pharmacological target, particularly in the context of inflammatory disorders [31]. However, developing medications that target ADAM17 has proven more difficult than anticipated. This is due to ADAM17's multifunctionality, which includes the release of approximately 90 other transmembrane proteins other than tumor necrosis factor (TNF), as well as its structural similarities to other metalloproteinases [31]. Increasing research suggest that E2 could modulate inflammatory mediators via its receptors (ERα and ERβ, GPER-1) during stress as a protective strategy. For instance, in ISO-induced stress state, studies have demonstrated that E2 supplementation could reverse and prevent cardiac inflammatory disorders via the downregulation of TNFα, a well-known substrate of ADAM17 [7]. Therefore, we speculated on the involvement of E2 in the adaptive regulation of ADAM17's expression during cardiac inflammation under stressful conditions.

A plethora of research shows that stress is associated with myocardial inflammation [7, 9, 12]. Indisputably, the postmortem examination of patients' hearts with stress-related diseases depicts abundant proinflammatory macrophages, which exacerbate myocardial proinflammatory responses [32, 33]. In conformity with our findings (Fig. 2), several studies have demonstrated a significant upregulation of inflammatory mediators and cardiac injury markers, along with notable cardiac tissue infiltration of macrophages and their association with E2 declination during stressful conditions [7, 9]. Also, studies have reported that E2 supplementation could reverse and prevent CVDs via the downregulation of inflammatory mediators, such as TNFα [15, 23]. This study demonstrated for the first time that E2 downregulated the RNA levels of myocardial ADAM17 along with the protein expressions of its two forms (pADAM17 and mADAM17) during ISO-induced stress in FVB female mice (Fig. 3). Also, we found a significant increase in the protein expression of the non-genomic receptor of E2, GPER-1, and its down signaling molecule, PI3K (Fig. 5A, B, C). Knocking out the GPER-1 gene in female C57BL/6 mice caused the upregulation of myocardial mADAM17 during stress. Additionally, the administration of the GPER-1 agonist, G1, significantly reduced the expression and infiltration of ADAM17 in the myocardia during ISO-induced stress (Fig. 4). Although ADAM17's physiological expression is optimal, its upregulation along with its substrates has been linked to several CVDs, indicating its crucial role in the development and progression of CVDs [9, 34–37], which is consistent with the results from this study. Recently, Wang et al. discovered a link between the mRNAs of the various E2 receptors and the mRNA of ADAM17, where the authors reported a positive association of GPER-1 with ADAM17 [38]. Taking it together, we anticipated that E2 may post-regulate the protein expressions or localization of ADAM17 via GPER-1 during stressful conditions, which needed further clarification.

As we embarked on this journey for further clarification, in vitro experiments in H9c2 cells revealed for the first time that E2 downregulated mADAM17's expression via the activation of GPER-1 and its down signaling molecule, PI3K. Thus, using G15 to antagonize GPER-1 activities in the presence of E2 resulted in the disappearance of E2's modulatory effect on mADAM17. However, the activation of GPER-1 with G1 brought back the modulatory effect of E2 (Fig. 5). GPER-1 is expressed in cardiomyocytes and governs the non-genomic cardioprotective role of E2 during myocardial injury [39]. Several pieces of evidence reveal that the activation of GPER-1 by E2 significantly reduces the release of proinflammatory cytokines, including TNFα [39–41], a keen substrate of mADAM17. For instance, a study by Zhang et al. demonstrated that the activation of GPER-1 by E2/G1 considerably decreases the release of proinflammatory cytokines such as TNF-α, IL-1β, and IL-6 during an ischemic injury in primary microglial cells [39]. Following GPER-1 activation, the upregulation of PI3K and its down signaling molecules, AKT and eNOS, has also been reported by many studies to be involved in the cardioprotective pathway of E2 [42, 43]. In line with this study, the results demonstrate that the cardioprotective effects of E2 were through the PI3K/AKT/eNOS signaling pathway. An attempt to inhibit the phosphorylation and activation of PI3K by LY294002 during ISO-induced stress resulted in the disappearance of the E2 modulatory effect on ADAM17 (Fig. 6).

It is well-known that in a stress state, elevated catecholamines result in the dysregulation of βARs, thus desensitization of β1ARs and overstimulation of β2ARs coupling with Gαi protein [7, 9, 16, 44]. Gαi signaling increases the activation of p38 MAPKs and ERKs [9, 15, 44], and phosphorylation by these kinases is a common way to significantly increase the membrane content and proteolytic activation of ADAM17 [9, 44–46]. For example, ERK-dependent threonine 735 (Thr735) phosphorylation is required for ADAM17 to enter the secretory route during maturation [9, 44, 45]. In addition, indirect ERKs or p38 MAPKs phosphorylation at 14–3-3 binding sites on the N-terminus of iRhoms turns on ADAM17's trafficking, stability, and cell surface expression [9, 44, 46]. Evidence also suggests that ADAM17's activities can be regulated by subcellular localization in the perinuclear region [45]. According to Peng et al., the protective role of PI3K/AKT/eNOS signaling in mechanical stress is via the inhibition of P38MAPKs in mice lungs [47]. However, no information exists on how E2 regulates myocardial ADAM17 in a stress state. A novel finding from this present study revealed that E2 via PI3K signaling downregulated mADAM17 through peri-nuclear localization by normalizing β1AR-Gαs expressions and mediating the switch from β2AR-Gαi to Gαs along with the reduction in phosphorylated ERKs and p38MAPKs (Fig. 7).

ADAM17 is a vital regulatory gateway in inflammation due to its tremendous proteolytic activity[44]. Indisputably, previous studies have implicated its upregulation along with its keen substrate, TNFα, in several inflammatory-associated diseases[7, 9, 36, 44]. Excessive liberation of TNFα has been shown to trigger its receptors, subsequently phosphorylating and activating NF-κB at the P65 subunit [9, 44, 48]. Active NF-κB can migrate into the nucleus to encode genes of proinflammatory cytokines, including IL-1β, which can ultimately result in inflammation [44, 49]. In this present study, we demonstrated for the first time that the downregulation of ADAM17 by E2 during stressful conditions could remarkably mitigate cardiac injury, decreasing the release of proinflammatory cytokines (TNFα, IL-1β) and significantly reducing the phosphorylation and activation of NF-κB at the P65 subunit in H9c2 cells (Fig. 8).

**Fig. 9:**
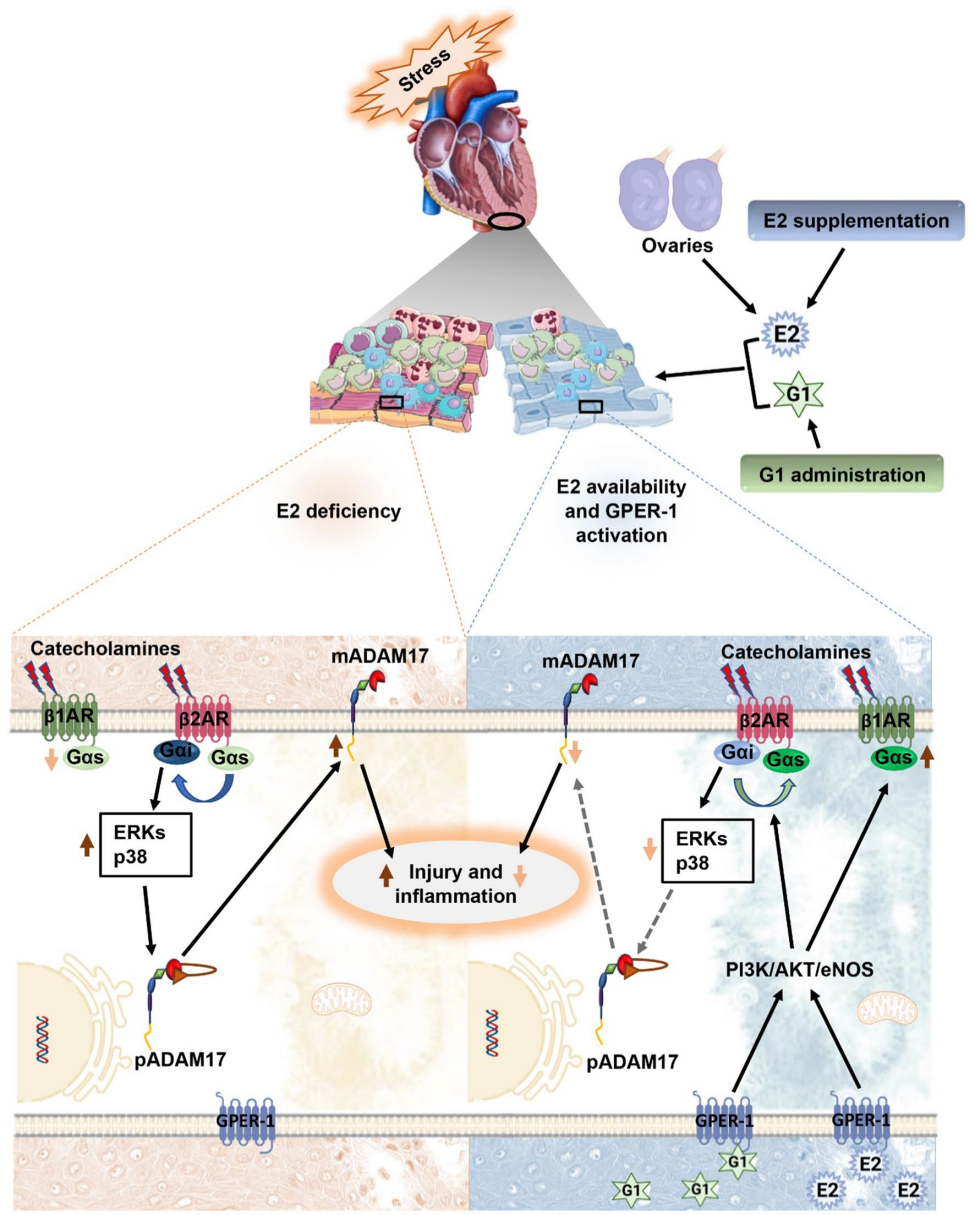
(Graphical abstract). A schematic diagram illustrating the effect of estradiol (E2) deficiency, its supplementation and G1 administration on ADAM17-induced injury and inflammation in the heart during a stressful condition. In a stress state, E2 deficiency enhances Gαi signaling and upregulates phosphorylated kinases (ERKs and p38 MAPKs), subsequently increasing ADAM17’s membrane content (mADAM17), which results in myocardial injury and inflammation. However, the availability of endogenous E2(produced by the ovaries) or exogenous E2 (E2 supplementation) mitigated ADAM17-induced myocardial injury and inflammation through the GPER-1/PI3K/AKT/eNOS pathway by mediating the switch from Gαi to Gαs signaling, hence decreasing phosphorylated kinases (ERKs and p38 MAPKs)

In conclusion, consistent with our findings, much evidence has demonstrated the cardioprotective effects of E2 via the modulation of inflammatory mediators during stressful situations, but the "how" remains a mystery. Overall, the schematics in Fig. 9 summarize the novel findings from this study, demonstrating for the first time that E2 attenuated stress-induced myocardial injury and inflammation via the modulation of ADAM17's expression. It also added to the body of knowledge on how E2 regulated ADAM17, thus, activating the GPER-1/PI3K/AKT/eNOS signaling pathway to localize this protease at the peri-nuclear regions, subsequently reducing its membrane content. Together, this research offers fresh insights into the physiological regulation of myocardial ADAM17 in females during stress. Additionally, from a pharmacological perspective, this study provides novel evidence that E2 and G1 are potential therapeutic agents for ADAM17-induced inflammatory diseases associated with postmenopausal females.

## Limitation

It is common for interesting studies to have limitations, and this study is no exception. While our study sheds light on the cardioprotective role of E2 via GPER-1 and its downstream signaling pathways, it is important to note that the complexity of the E2 signaling network means that our findings are just the tip of the iceberg. Although we focused on GPER-1 due to its rapid membrane-mediated effects, we could not capture a more comprehensive picture of E2’s genomic receptors' involvement, which could be accomplished by inhibiting them. Importantly, our findings revealed that E2 treatment during stress reduced ADAM17’s mRNA levels in the myocardium, implying the involvement of E2’s genomic receptors; hence future studies should explore the potential involvement of ERα and ERβ in mediating E2's cardioprotective effects via the modulation of ADAM17 during stress. Understanding the multifaceted mechanisms of E2 signaling in cardiovascular stress could provide valuable insights for developing novel therapies for cardiovascular diseases, which remain a leading cause of morbidity and mortality worldwide.

## Data Availability

The authors confirmed that all data required to evaluate the study's conclusions are present in the paper.

## Abbreviations

ADAM17: A Disintegrin and Metalloprotease 17
ANOVA: Analysis of variance
βARs: β-Adrenergic receptors
BSA: Bovine serum albumin
CD: Cluster of differentiation
CVDs: Cardiovascular diseases
E2: Estradiol
ERα: Estrogen receptor alpha
ERβ: Estrogen receptor beta
ERKs: Extracellular signal-regulated kinases
GPCRs: G protein-coupled receptors
GPER-1: G protein-coupled estradiol receptor-1
IL: Interleukin
ISO: Isoproterenol
OVX: Ovariectomized
p38 MAPKs: P38 mitogen-activated-protein-kinases
PI3K: Phosphoinositide 3 kinase
SEM: Standard error mean
TNF-α: Tumor necrosis factor alpha
WGA: Wheat germ agglutinin
